# Publisher Correction: Probing the strongly driven spin-boson model in a superconducting quantum circuit

**DOI:** 10.1038/s41467-018-04348-9

**Published:** 2018-06-07

**Authors:** L. Magazzù, P. Forn-Díaz, R. Belyansky, J.-L. Orgiazzi, M. A. Yurtalan, M. R. Otto, A. Lupascu, C. M. Wilson, M. Grifoni

**Affiliations:** 1 Institute of Physics, University of Augsburg, Universitätsstraße 1, D-86135 Augsburg, Germany; 2 Institute for Quantum Computing, University of Waterloo, N2L 3G1 Waterloo, Canada; 3 Department of Physics and Astronomy, University of Waterloo, N2L 3G1 Waterloo, Canada; 4 Waterloo Institute for Nanotechnology, University of Waterloo, N2L 3G1 Waterloo, Canada; 5 Barcelona Supercomputing Center (BSC), C/Jordi Girona 29, 08034 Barcelona, Spain; 6 Department of Electrical and Computer Engineering, University of Waterloo, N2L 3G1 Waterloo, Canada; 7 Institute for Theoretical Physics, University of Regensburg, 93040 Regensburg, Germany

Correction to: *Nature Communications* 10.1038/s41467-018-03626-w, published online 11 April 2018.

The original PDF and HTML versions of this Article omitted the ORCID ID of the authors L. Magazzù and P. Forn-Díaz. (L. Magazzù: 0000-0002-4377-8387; P. Forn-Diaz: 0000-0003-4365-5157)

The original PDF version of this Article contained errors in Eqs. (2), (6), (13), (14), (25), (26). These equations were missing all instances of ‘*Γ*’ and ‘Δ’, which are correctly displayed in the HTML version.

Similarly, the third sentence in the legend of Fig. 2 originally incorrectly read ‘The position ω* and FWHM 2γ of the linear susceptibility peak in the coherent regimes (*α* = 0.007, *α* = 0.21) provide a direct measure of the renormalized qubit frequency $= \sqrt {\left( {\omega ^ \ast } \right)^2 - \gamma ^2}$.’ The correct version states ‘$\Omega = \sqrt {\left( {\omega ^ \ast } \right)^2 - \gamma ^2}$’ instead of ‘$= \sqrt {\left( {\omega ^ \ast } \right)^2 - \gamma ^2}$’.

The original HTML version of this Article contained errors in Table 1. The correct version of the sixth row of the first column states ‘Figure 2’ instead of the original, incorrect ‘Figure’. And the correction version of the ninth row of the first column states ‘Figure 3’ instead of the original, incorrect ‘Figure’.

This has been corrected in both the PDF and HTML versions of the Article.

