# Supplementary material for: Probing the strongly driven spin-boson model in a superconducting quantum circuit
Source: Nat Commun. 2018 Apr 11;9:1403. doi: 10.1038/s41467-018-03626-w (PMC5895759; doi:10.1038/s41467-018-03626-w)
Supplement: Supplementary file 1 — Supplementary Information [file 41467_2018_3626_MOESM1_ESM.pdf]

Supplementary Information – Probing the strongly driven spin-boson model in a superconducting quantum circuit

L. Magazzù *et al.*

Supplementary note 1: Generalized master equation for the driven spin-boson model

The spin-boson model describes a two-level system – the qubit – interacting with an environment of quantum harmonic oscillators, the so-called heat bath.
The total Hamiltonian of the model reads

$$H(t) = -\frac{\hbar}{2}\left[\Delta\sigma_x + \varepsilon(t)\sigma_z\right] - \frac{\hbar}{2}\sigma_z \sum_i c_i(a_i^\dagger + a_i) + \sum_i \hbar\omega_i a_i^\dagger a_i \ , \tag{1}$$

where σ_j are Pauli spin operators and $a_i^\dagger$ and a_i are bosonic creation and annihilation operators, respectively. The angular frequency Δ is the bare frequency splitting at zero bias. Within the noninteracting-blip approximation (NIBA), the time evolution of the qubit's population difference $P(t) = \langle\sigma_z(t)\rangle$ is governed by the following generalized master equation (GME) [1–3]

$$\dot{P}(t) = \int_{t_0}^{t} dt' \left[\mathcal{K}^-(t,t') - \mathcal{K}^+(t,t')P(t')\right] \ . \tag{2}$$

In the presence of a time dependent bias described by $\varepsilon(t) = \varepsilon_0 + \varepsilon_{\rm p}\cos(\omega_{\rm p}t) + \varepsilon_{\rm d}\cos(\omega_{\rm d}t)$, where the subscripts "p" and "d" denote probe and drive, respectively, the exact NIBA kernels are

$$\mathcal{K}_{\rm N}^+(t,t') = \Delta^2 e^{-Q'(t-t')}\cos[Q''(t-t')]\cos\left[\zeta_{\rm tot}(t,t')\right] \ , \tag{3}$$

$$\mathcal{K}_{\rm N}^-(t,t') = \Delta^2 e^{-Q'(t-t')}\sin[Q''(t-t')]\sin\left[\zeta_{\rm tot}(t,t')\right] \ , \tag{4}$$

where the total dynamical phase has the form

$$\zeta_{\rm tot}(t,t') = \int_{t'}^{t} dt'' \ \varepsilon(t'') \ . \tag{5}$$

Averaging over a period $2\pi/\omega_{\rm d}$ yields an effective description of the drive by means of the following NIBA kernels [3], which we use for our calculations

$$\mathcal{K}^+(t,t') = h^+(t-t')\cos\left[\zeta(t,t')\right] \ , \tag{6}$$

$$\mathcal{K}^-(t,t') = h^-(t-t')\sin\left[\zeta(t,t')\right] \ , \tag{7}$$

with the functions $h^{\pm}(t)$ reading

$$h^{+}(t) = \Delta^2 e^{-Q'(t)} \cos[Q''(t)] J_0 \left[\frac{2\varepsilon_{\rm d}}{\omega_{\rm d}} \sin\left(\frac{\omega_{\rm d} t}{2} \right) \right] \ , \tag{8}$$

$$h^{-}(t) = \Delta^2 e^{-Q'(t)} \sin[Q''(t)] J_0 \left[\frac{2\varepsilon_{\rm d}}{\omega_{\rm d}} \sin\left(\frac{\omega_{\rm d} t}{2} \right) \right] \ . \tag{9}$$

The dynamical phase

$$\zeta(t,t') = \varepsilon_0 (t - t') + \frac{\varepsilon_{\rm p}}{\omega_{\rm p}} \left[\sin(\omega_{\rm p} t) - \sin\left(\omega_{\rm p} t'\right) \right] \tag{10}$$

entering the averaged NIBA kernels in Supplementary Equations (6)-(7) accounts now exclusively for the static bias and the probe field, whereas the drive is taken into account, in an effective description, by the Bessel functions J_0 in the functions of $h^{\pm}(t)$.

The functions Q' and Q'' in Supplementary Equations (3)-(4) and (8)-(9), are the real and imaginary part of the bath correlation function $Q(t)$, respectively. For Ohmic spectral density function $G(\omega) = 2\alpha\omega \exp(-\omega/\omega_{\rm c})$, α being the dimensionless coupling strength and $\omega_{\rm c}$ a cutoff frequency, these two functions have the following explicit expressions [1]

$$Q'(t) = \alpha \ln(1 + \omega_{\rm c}^2 t^2) + 4\alpha \ln \left| \frac{\Gamma(1 + \omega_\beta/\omega_{\rm c})}{\Gamma(1 + \omega_\beta/\omega_{\rm c} + {\rm i}\omega_\beta t)} \right| \ , \tag{11}$$

$$Q''(t) = 2\alpha \arctan(\omega_{\rm c} t) \ , \tag{12}$$

where we have introduced the thermal frequency $\omega_\beta = (\hbar\beta)^{-1}$ and where $\Gamma(x)$ is the Euler Gamma function. In the limit $\hbar\omega_{\rm c} \gg k_{\rm B}T$ (or $\omega_{\rm c} \gg \omega_\beta$), neglecting the ratio $\omega_\beta/\omega_{\rm c}$ and using $\Gamma(1 + {\rm i}x)\Gamma(1 - {\rm i}x) = \pi x / \sinh(\pi x)$, we get the so-called scaling limit forms

$$Q'(t) = 2\alpha \ln \left[\sqrt{1 + \omega_c^2 t^2} \frac{\sinh(\pi\omega_\beta t)}{\pi\omega_\beta t} \right] \ , \tag{13}$$

$$Q''(t) = 2\alpha \arctan(\omega_c t) \ . \tag{14}$$

These expressions are accurate in every regime, provided that the cutoff frequency is large with respect to the other frequency scales involved. For $\omega_{\rm c} t \gg 1$, these functions assume the approximated forms

$$Q'(t) \simeq 2\alpha \ln \left[\frac{\omega_{\rm c}}{\pi\omega_\beta} \sinh(\pi\omega_\beta t) \right] \ , \tag{15}$$

$$Q''(t) \simeq \pi\alpha {\rm sgn}(t) \ . \tag{16}$$

Especially at high temperature, $\omega_\beta \sim \Delta$, the cutoff operated by the real part $Q'(t)$ in the kernels, becomes of purely exponential form on a short time scale, see Supplementary Equation (17) below. Now, this means that, at strong coupling, the kernels go to zero on a rather short time, where the short time behavior of Q'', neglected in Supplementary Equation (16), is relevant. Therefore we will use the approximated expressions in Supplementary Equations (15)-(16) only for $\alpha < 0.5$.

An insight into the different behaviors shown by the two driven setups in Fig. 3 of the main text, is provided by considering the memory time of the kernels $\mathcal{K}^{\pm}$. To this end, consider the long-time limit of $Q(t)$ in Supplementary Equations (15)-(16). Specifically, for $\omega_\beta t = t k_{\rm B}T/\hbar \gg 1$, the real part of $Q(t)$ acquires the form

$$Q'(t) \sim t/\tau_{\rm env} + \text{const.}, \qquad \text{where} \qquad \tau_{\rm env} = (2\pi\alpha k_{\rm B}T/\hbar)^{-1} \,. \tag{17}$$

This form implies that, at fixed, finite temperature, $\tau_{\rm env}$ decreases as the coupling α is increased. Moreover, in the above limit, the bath force operator $F(t)$ of the quantum Langevin equation for the spin-boson model is delta-correlated, as $\langle F(t)F(0)\rangle \propto \frac{d^2}{dt^2}Q(|t|)$, where the average is taken with respect to the thermal state of the bath (see Ref. [1] for details). As a consequence, on the time scale dictated by the limit (17) the bath is a white noise source.

Supplementary Note 2: Relating the transmission to the qubit's dynamics

Consider the situation depicted in Supplementary Figure 1, in which the probe voltage field $V_{\rm p}^{\rm in}(t) = f_{\rm Z}\varepsilon_{\rm p}\cos(\omega_{\rm p}t)$, coming from the left, is scattered by the qubit placed at the center of the transmission line. The proportionality constant $f_{\rm Z}$ has dimensions of flux whereas $\varepsilon_{\rm p}$ is an angular frequency. The scattering at the qubit position results in the transmitted field to the right, $V_{\rm transm}(t)$, and a reflected field to the left, $V_{\rm refl}(t)$. The flux difference across the qubit is $\delta\Phi(t) = \Phi^{\rm L}(t) - \Phi^{\rm R}(t)$, the flux being related to the voltage by $\Phi(t) = \int_{-\infty}^{t} dt'\, V(t')$.

A discretized circuit model [4] with inductance and capacitance per unit length l and c, respectively, gives for the voltage $V(0^-,t) \equiv V^{\rm L}(t)$ and current $I(0^-,t) \equiv I^{\rm L}(t)$ immediately to

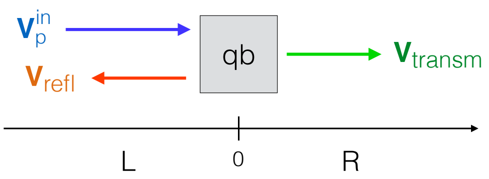

Supplementay Figure 1. Reflection and transmission of the incoming voltage $V_{\rm p}^{\rm in}$.

the left of the qubit the following equations

$$V^{\rm L}(t) = V_{\rm p}^{\rm in}(t) + V_{\rm refl}(t) \ , \tag{18}$$

$$I^{\rm L}(t) = \frac{1}{Z}\left[V_{\rm p}^{\rm in}(t) - V_{\rm refl}(t)\right] \ , \tag{19}$$

where $Z = \sqrt{l/c}$ is the characteristic impedance of the transmission line. Similarly, to the right of the qubit, where we set $V(0^+, t) \equiv V^{\rm R}(t)$ and $I(0^+, t) \equiv I^{\rm R}(t)$, we have

$$V^{\rm R}(t) = V_{\rm transm}(t) \ , \tag{20}$$

$$I^{\rm R}(t) = \frac{1}{Z} V_{\rm transm}(t) \ . \tag{21}$$

Using the conservation of the current, $I^{\rm L}(t) = I^{\rm R}(t)$, and the relation $V^{\rm L}(t) - V^{\rm R}(t) = \delta\dot{\Phi}(t)$, from Supplementary Equations (18)-(21) we get

$$V_{\rm transm}(t) = V_{\rm p}^{\rm in}(t) - \frac{\delta\dot{\Phi}(t)}{2} \ . \tag{22}$$

We identify the flux difference across the qubit with the population difference of the localized eigenstates of the flux operator $\hat{\Phi} = f\sigma_z$, namely we set $\delta\Phi(t) \equiv f\langle\sigma_z(t)\rangle = fP(t)$, where f is the proportionality constant with dimensions of flux, as described in the main text.

Let $P^{\rm as}(t) = \lim_{t\to\infty} P(t)$ be the asymptotic, nonequilibrium population difference. For periodic driving with period $2\pi/\omega_{\rm p}$, the time derivative $\dot{P}^{\rm as}(t)$ can be expanded as the Fourier series

$$\dot{P}^{\rm as}(t) = \sum_m {\rm i}m\omega_{\rm p} p_m e^{{\rm i}m\omega_{\rm p}t} \ , \tag{23}$$

where

$$p_m = \frac{\omega_{\rm p}}{2\pi}\int_{-\pi/\omega_{\rm p}}^{\pi/\omega_{\rm p}} dt \ P^{\rm as}(t) e^{-{\rm i}m\omega_{\rm p}t} \ . \tag{24}$$

The transmission $\mathcal{T}$ at frequency ω_p ($m = 1$) is defined as the following ratio between transmitted and input voltages

$$\begin{aligned}\mathcal{T}(\omega_\mathrm{p}) &= \frac{V_\mathrm{transm}(\omega_\mathrm{p})}{V_\mathrm{p}^\mathrm{in}(\omega_\mathrm{p})} \\ &= \frac{f_\mathrm{Z}\varepsilon_\mathrm{p}/2 - \mathrm{i}f\omega_\mathrm{p}p_1/2}{f_\mathrm{Z}\varepsilon_\mathrm{p}/2} \\ &= 1 - \mathrm{i}\mathcal{N}\omega_\mathrm{p}p_1/\varepsilon_\mathrm{p}\ ,\end{aligned} \tag{25}$$

where $\mathcal{N} = f/f_\mathrm{Z}$ and where, in passing from the first to the second line, we used Supplementary Equations (22) and (23). Real and imaginary parts of the transmission are therefore given by

$$\mathrm{Re}\{\mathcal{T}(\omega_\mathrm{p})\} = 1 + \mathcal{N}\omega_\mathrm{p}\mathrm{Im}\{p_1\}/\varepsilon_\mathrm{p} \tag{26}$$

$$\text{and} \qquad \mathrm{Im}\{\mathcal{T}(\omega_\mathrm{p})\} = -\mathcal{N}\omega_\mathrm{p}\mathrm{Re}\{p_1\}/\varepsilon_\mathrm{p}\ , \tag{27}$$

respectively.

Supplementary Note 3: Linear response to a weak probe – closed expression for the transmission

In the regime of linear response to an applied monochromatic probe driving, namely for small ratio $\varepsilon_\mathrm{p}/\omega_\mathrm{p}$, and within the effective description of the pump drive introduced in the Supplementary Note 1, the asymptotic population difference $P^\mathrm{as}(t)$ is monochromatic [3, 5]. It can be thus expressed as the truncated Fourier sum

$$\begin{aligned}P^\mathrm{as}(t) &\simeq p_0 + p_1^{(1)}e^{\mathrm{i}\omega_\mathrm{p}t} + p_{-1}^{(1)}e^{-\mathrm{i}\omega_\mathrm{p}t} \\ &= P_0 + \hbar\varepsilon_\mathrm{pr}[\chi(\omega_\mathrm{p})e^{\mathrm{i}\omega_\mathrm{p}t} + \chi(-\omega_\mathrm{p})e^{-\mathrm{i}\omega_\mathrm{p}t}]\ ,\end{aligned} \tag{28}$$

where the superscript $^{(1)}$ denotes first order with respect to the ratio $\varepsilon_\mathrm{p}/\omega_\mathrm{p}$. Here χ is the linear susceptibility [5] and P_0 is the asymptotic value of $P(t)$ in absence of probe driving. As shown in Supplementary Figure 2 below, this constitutes an excellent approximation of the actual dynamics under weak probe driving. From Supplementary Equations (25) and (28), the transmission at probe frequency in linear response is related to the dynamical susceptibility by

$$\mathcal{T}(\omega_\mathrm{p}) = 1 - \mathrm{i}\mathcal{N}\hbar\omega_\mathrm{p}\chi(\omega_\mathrm{p})\ . \tag{29}$$

Within the NIBA, by substituting the expression (28) for $P^{\rm as}(t)$ in the GME (2), setting the upper integration limit to $t \to \infty$, which is valid for times much larger than the kernels' memory time, and expanding the kernels in Fourier series, we get the following closed, linear response expression for $p_1^{(1)}$ [3, 5]

$$p_1^{(1)}(\omega_{\rm p}) = \frac{1}{{\rm i}\omega_{\rm p} + v^{+(0)}(\omega_{\rm p})}\left[k_1^{-(1)}(\omega_{\rm p}) - k_1^{+(1)}(\omega_{\rm p})\frac{k_0^{-(0)}}{k_0^{+(0)}}\right] \tag{30}$$

(superscripts $^{(0,1)}$ denote the order in $\varepsilon_{\rm p}/\omega_{\rm p}$).

The kernels $k_m^{\pm}$ and v^{+}, whose approximate forms (perturbative in $\varepsilon_{\rm p}/\omega_{\rm p}$) enter Supplementary Equation (30), are defined by

$$k_m^{\pm}(\omega_{\rm p}) = \frac{\omega_{\rm p}}{2\pi}\int_{-\pi/\omega_{\rm p}}^{\pi/\omega_{\rm p}} dt\; e^{-{\rm i}m\omega_{\rm p}t}\int_0^{\infty} d\tau\; \mathcal{K}^{\pm}(t, t-\tau)\;, \tag{31}$$

$$v^{+}(\omega_{\rm p}) = \frac{\omega_{\rm p}}{2\pi}\int_{-\pi/\omega_{\rm p}}^{\pi/\omega_{\rm p}} dt\; \int_0^{\infty} d\tau\; e^{-{\rm i}\omega_{\rm p}\tau}\mathcal{K}^{\pm}(t, t-\tau)\;, \tag{32}$$

$$\tag{33}$$

where the pump drive-averaged kernels $\mathcal{K}^{\pm}(t, t')$ have been introduced in Supplementary Equations (6)-(7). Expansion of the Bessel functions entering the kernels $\mathcal{K}^{\pm}(t, t')$ to lowest order in $\varepsilon_{\rm p}/\omega_{\rm p}$ by means of $J_n(x) \sim (x/2)^n$, yields the following explicit expressions for the kernels in Supplementary Equation (30)

$$k_0^{+(0)} = \int_0^{\infty} dt\; h^{+}(t)\cos(\varepsilon_0 t)\;, \tag{34}$$

$$k_0^{-(0)} = \int_0^{\infty} dt\; h^{-}(t)\sin(\varepsilon_0 t)\;, \tag{35}$$

$$k_1^{+(1)}(\omega_{\rm p}) = -\frac{\varepsilon_{\rm p}}{\omega_{\rm p}}\int_0^{\infty} dt\; e^{-{\rm i}\omega_{\rm p}t/2}h^{+}(t)\sin(\varepsilon_0 t)\sin(\omega_{\rm p}t/2)\;, \tag{36}$$

$$k_1^{-(1)}(\omega_{\rm p}) = \frac{\varepsilon_{\rm p}}{\omega_{\rm p}}\int_0^{\infty} dt\; e^{-{\rm i}\omega_{\rm p}t/2}h^{-}(t)\cos(\varepsilon_0 t)\sin(\omega_{\rm p}t/2)\;, \tag{37}$$

$$\text{and}\quad v^{+(0)}(\omega_{\rm p}) = \int_0^{\infty} dt\; e^{-{\rm i}\omega_{\rm p}t}h^{+}(t)\cos(\varepsilon_0 t)\;, \tag{38}$$

with $h^{\pm}(t)$ defined in Supplementary Equations (8)-(9). In Supplementary Figure 2 the transient dynamics obtained from direct integration of the GME (2) is compared to the asymptotic time-periodic evolution given by Supplementary Equations (28), (30), and (34)-(38).

The linear susceptibility χ is related to the coefficient $p_1^{(1)}$ by Supplementary Equation (28).

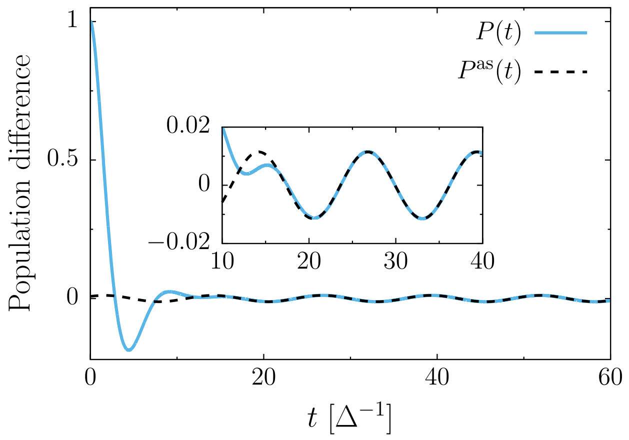

Supplementay Figure 2. Linear response to a weak probe field – dynamics of the undriven qubit. Time evolution of the population difference $P(t)$ obtained by integrating the GME (2) with $P(0) = 1$ (solid line) compared with the asymptotic dynamics $P^{\rm as}(t)$ given by Supplementary Equation (28) with $p_1^{(1)}$ from Supplementary Equation (30) (dashed line). The kernels $k_m^{\pm}$ and v^+ are obtained by numerically evaluating the integrals in Supplementary Equations (34)-(38). The bath correlation function $Q(t)$ in exact scaling limit form [Supplementary Equations (13)-(14)] is used for both curves. Parameters are $\alpha = 0.2$, $T = 0.5\ \hbar\Delta/k_{\rm B}$, $\omega_{\rm c} = 10\ \Delta$, $\varepsilon_0 = 0$, $\varepsilon_{\rm d} = 0$, $\omega_{\rm p} = 0.5\ \Delta$, and $\varepsilon_{\rm p} = 0.01\ \Delta$.

Thus, from Supplementary Equation (30), by simplifying the notation, we get

$$\chi(\omega_{\rm p}) = \frac{H^+(\omega_{\rm p}) - H^-(\omega_{\rm p})P_0}{{\rm i}\omega_{\rm p} + K^+({\rm i}\omega_{\rm p})}\ , \qquad \text{where} \qquad P_0 = K^-(0)/K^+(0)\ . \tag{39}$$

Here $K^{\pm}(\lambda) = \int_0^\infty d\tau e^{-\lambda\tau}\mathcal{K}^{\pm}(\tau)$ is the Laplace transform of the pump-averaged kernels with $\varepsilon_{\rm p} = 0$. The kernels in Supplementary Equation (39) are related to the ones defined in Supplementary Equations (34)-(38) by

$$K^{\pm}(\lambda = 0) = k_0^{\pm(0)}\ , \quad K^+(\lambda = {\rm i}\omega_{\rm p}) = v^{+(0)}(\omega_{\rm p})\ , \quad \text{and} \quad H^{\pm}(\omega_{\rm p}) = \frac{k_1^{\mp(1)}(\omega_{\rm p})}{\hbar\varepsilon_{\rm p}}\ . \tag{40}$$

Note that, within the present linear response treatment, the transmission is independent of the probe amplitude $\varepsilon_{\rm p}$, cf. Supplementary Equation (29). Note also that the notation for the kernels $H^{\pm}$ reflects the same symmetry with respect to the static bias ε_0 which holds for $K^{\pm}$.

Finally, the forward/backward rates

$$K^{\mathrm{f/b}} = [K^+(0) \pm K^+(0)]/2$$
$$= \frac{\Delta^2}{2} \int_0^\infty dt\, e^{-Q'(t)} J_0 \left[\frac{2\varepsilon_{\mathrm{d}}}{\omega_{\mathrm{d}}} \sin\left(\frac{\omega_{\mathrm{d}} t}{2}\right)\right] \cos[Q''(t) \mp \varepsilon_0 t] \;, \tag{41}$$

introduced in the main text, describe the incoherent tunneling between the individual localized (flux) states.

Supplementary Note 4: Approximate form of the susceptibility

Whenever the condition $\omega_{\mathrm{p}}\tau_{\mathrm{env}} \ll 1$ is fulfilled, it is possible to expand the kernels $K^+(\mathrm{i}\omega_{\mathrm{p}})$ and $H^\pm(\omega_{\mathrm{p}})$ [see Supplementary Equation (40)] with respect to $\omega_{\mathrm{p}}\tau_{\mathrm{env}}$. To first order

$$K^+(\mathrm{i}\omega_{\mathrm{p}}) \simeq K^+(\lambda = 0) \qquad \text{and} \qquad H^\pm(\omega_{\mathrm{p}}) \simeq \frac{1}{2\hbar} \frac{\partial}{\partial \varepsilon_0} K^\mp(\lambda = 0) \;. \tag{42}$$

Now, the NIBA prediction for the stationary probability difference P_0 in the absence of the pump driving is $P_0 = \tanh(\hbar\varepsilon_0/2k_{\mathrm{B}}T)$ [1, 3]. In the presence of the pump driving, within the present effective description of the pump drive (see the Supplementary Note 1), the expression for P_0 is generalized as follows

$$P_0 = \tanh\left(\frac{\hbar\varepsilon_{\mathrm{eff}}}{2k_{\mathrm{B}}T}\right) \qquad \text{where} \qquad \varepsilon_{\mathrm{eff}} = \frac{k_{\mathrm{B}}T}{\hbar} \ln\left(\frac{K^{\mathrm{f}}}{K^{\mathrm{b}}}\right) \;. \tag{43}$$

The effective bias $\varepsilon_{\mathrm{eff}}$ depends on the static bias ε_0. As a result, in the limit $\omega_{\mathrm{p}}\tau_{\mathrm{env}} \ll 1$, by substituting the expressions in Supplementary Equation (42) into Supplementary Equation (39) we obtain

$$\chi(\omega_{\mathrm{p}}) \simeq \frac{K^+(0)}{2\hbar[\mathrm{i}\omega_{\mathrm{p}} + K^+(0)]} \frac{\partial}{\partial\varepsilon_0} \tanh\left(\frac{\hbar\varepsilon_{\mathrm{eff}}}{2k_{\mathrm{B}}T}\right)$$
$$= \frac{1}{4k_{\mathrm{B}}T} \frac{\partial\varepsilon_{\mathrm{eff}}/\partial\varepsilon_0}{\cosh^2(\hbar\varepsilon_{\mathrm{eff}}/2k_{\mathrm{B}}T)} \frac{\gamma_{\mathrm{d}}}{\gamma_{\mathrm{d}} + \mathrm{i}\omega_{\mathrm{p}}} \;, \tag{44}$$

where $\gamma_{\mathrm{d}} = K^+(0) = K^{\mathrm{f}} + K^{\mathrm{b}}$ (cf. Supplementary Equation 41), and where

$$\frac{\partial\varepsilon_{\mathrm{eff}}}{\partial\varepsilon_0} = \frac{k_{\mathrm{B}}T}{\hbar} \left(\frac{1}{K^{\mathrm{f}}} \frac{\partial K^{\mathrm{f}}}{\partial\varepsilon_0} - \frac{1}{K^{\mathrm{b}}} \frac{\partial K^{\mathrm{b}}}{\partial\varepsilon_0}\right) \;. \tag{45}$$

At the symmetry point $\varepsilon_{\mathrm{eff}} = \varepsilon_0 = 0$ so that from Supplementary Equations (41) and (45) we get

$$\lim_{\varepsilon_0 \to 0} \frac{\partial\varepsilon_{\mathrm{eff}}}{\partial\varepsilon_0} = \frac{2k_{\mathrm{B}}T}{\hbar} \frac{\int_0^\infty dt\, t\, h^-(t)}{\int_0^\infty dt\, h^+(t)} \;, \tag{46}$$

where the functions $h^\pm(t)$ have been defined in Supplementary Equations (8)-(9).

Supplementary Note 5: Analytical evaluation of the kernels in the absence of pump driving

The integrals in Supplementary Equations (34)-(38) can be solved analytically by using the bath correlation function $Q(t)$ in the approximated scaling limit form given by Supplementary Equations (15)-(16) for $\alpha < 1/2$. With this approximated correlation function, the functions $h^{\pm}(t)$, introduced in Supplementary Equations (8)-(9), take on the form

$$h^{+}(t) = \Delta^2 (2\kappa\omega_{\rm c})^{-2\alpha} \left[\sinh(t/2\kappa)\right]^{-2\alpha} \cos(\pi\alpha) \, , \tag{47}$$

$$h^{-}(t) = \Delta^2 (2\kappa\omega_{\rm c})^{-2\alpha} \left[\sinh(t/2\kappa)\right]^{-2\alpha} \sin(\pi\alpha) \, , \tag{48}$$

where we introduced the time scale $\kappa = \hbar\beta/2\pi = (2\pi\omega_\beta)^{-1}$.

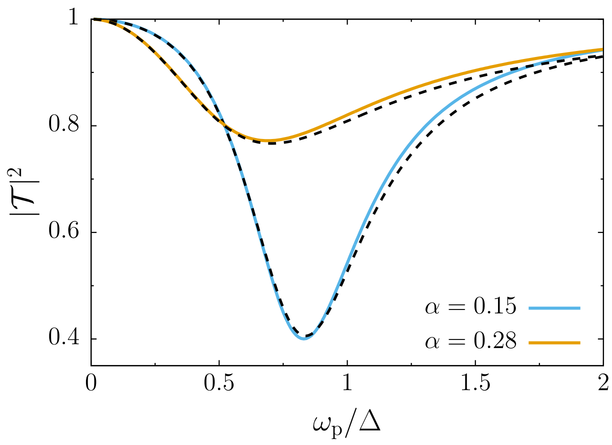

Supplementay Figure 3. Transmission *vs.* probe frequency for the undriven qubit for two values of α. The transmission is calculated via Supplementary Equations (29) and (39). Solid lines – kernels numerically evaluated from Supplementary Equations (34)-(38) with the bath correlation function $Q(t)$ in the exact scaling limit from [Supplementary Equations (13)-(14)]. Dashed lines – kernels in analytical approximated forms [Supplementary Equations (51)-(54)]. Parameters are $T = 0.5\ \hbar\Delta/k_{\rm B}$, $\omega_{\rm c} = 10\ \Delta$, $\varepsilon_0 = 0$, $\varepsilon_{\rm d} = 0$, and $\varepsilon_{\rm p} = 0.01\ \Delta$.

We use the exact result [6]

$$\int_0^\infty dt\, e^{-\mu t} \sinh^{\nu}(\beta t) = \frac{1}{2^{\nu+1}\beta} B\left(\frac{\mu}{2\beta} - \frac{\nu}{2}, \nu + 1\right) , \tag{49}$$

where $B(x,y)$ is the beta function with the property

$$B(x,y)=\frac{\Gamma(x)\Gamma(y)}{\Gamma(x+y)}, \tag{50}$$

and $\Gamma(z)$ is the Euler Gamma function, with the property $\Gamma(1-z)\Gamma(z)=\pi/\sin(\pi z)$. By setting $\mu=\mathrm{i}(\omega_\mathrm{p}\pm\varepsilon_0)$, $\nu=-2\alpha$, and $\beta=(2\kappa)^{-1}$, we obtain the following analytical expression for the kernels in Supplementary Equation (40)

$$K^+(\lambda)=N_+\left[\mathcal{W}(-\mathrm{i}\lambda+\varepsilon_0)+\mathcal{W}(-\mathrm{i}\lambda-\varepsilon_0)\right]\ , \tag{51}$$

$$K^-(0)=\mathrm{i}N_-\left[\mathcal{W}(\varepsilon_0)-\mathcal{W}(-\varepsilon_0)\right]\ , \tag{52}$$

$$H^-(\omega_\mathrm{p})=\frac{1}{2\hbar\omega_\mathrm{p}}N_+\left[\mathcal{W}(\omega_\mathrm{p}+\varepsilon_0)-\mathcal{W}(\omega_\mathrm{p}-\varepsilon_0)-\mathcal{W}(\varepsilon_0)+\mathcal{W}(-\varepsilon_0)\right]\ , \tag{53}$$

$$\text{and}\quad H^+(\omega_\mathrm{p})=\mathrm{i}\frac{\varepsilon_\mathrm{p}}{2\omega_\mathrm{p}}N_-\left[\mathcal{W}(\omega_\mathrm{p}+\varepsilon_0)+\mathcal{W}(\omega_\mathrm{p}-\varepsilon_0)-\mathcal{W}(\varepsilon_0)-\mathcal{W}(-\varepsilon_0)\right]\ , \tag{54}$$

where

$$N_+=\frac{\Delta^2}{2}\frac{\kappa^{1-2\alpha}}{\omega_\mathrm{c}^{2\alpha}}\cos(\pi\alpha)\Gamma(1-2\alpha)\ , \tag{55}$$

$$N_-=\frac{\Delta^2}{2}\frac{\kappa^{1-2\alpha}}{\omega_\mathrm{c}^{2\alpha}}\sin(\pi\alpha)\Gamma(1-2\alpha)\ , \tag{56}$$

$$\text{and}\quad \mathcal{W}(x)=\frac{\Gamma(\alpha+\mathrm{i}\kappa x)}{\Gamma(1-\alpha+\mathrm{i}\kappa x)}\ . \tag{57}$$

Note that $\mathcal{W}(-x)=\mathcal{W}^*(x)$. In Supplementary Figure 3, the transmission obtained by using the analytical expressions in Supplementary Equations (51)-(54) is compared with the corresponding numerical evaluations of Supplementary Equations (34)-(38) with $Q(t)$ in the exact scaling limit form [Supplementary Equations (13)-(14)].

The analytical expressions in Supplementary Equations (51)-(54) are used to perform fits to data (see the next section) and for the theory colormaps for devices I and II in Fig. 2 of the main text ($\alpha=0.007$ and 0.21, respectively, and $\varepsilon_\mathrm{d}=0$). This is not the case for Device III in the same figure ($\alpha=0.8$, $\varepsilon_\mathrm{d}=0$) and for the theory panels in Fig. 3 ($\varepsilon_\mathrm{d}\neq 0$) of the main text, where the transmission is calculated by numerically evaluating the integrals in Supplementary Equations (34)-(38) with correlation function $Q(t)$ in the exact scaling limit form.

Supplementary Note 6: Fit to data for devices I and II in the absence of pump driving

In Supplementary Figure 4 we show the results of fits to the measured transmission at the symmetry point ($\Phi_\epsilon = \Phi_0/2$, where Φ_ϵ is the control field associated with the static bias) for the devices I and II. The spectra of these devices in the absence of drive are shown in Fig. 2 of the main text. Fits to data are performed by using Supplementary Equations (29) and (39) with the analytical expressions in Supplementary Equations (51)-(54) for the kernels $K^{\pm}$ and $H^{\pm}$. Note that the present treatment has as input the bare value of Δ, the qubit splitting at zero bias, which is not accessed directly in experiments. For this reason Δ is left as a free parameter, along with the spin-boson coupling α and the prefactor $\mathcal{N}$ in Supplementary Equation (29). Temperature and cutoff frequency are fixed to $T = 90$ mK and $\omega_c/2\pi = 65$ GHz, respectively.

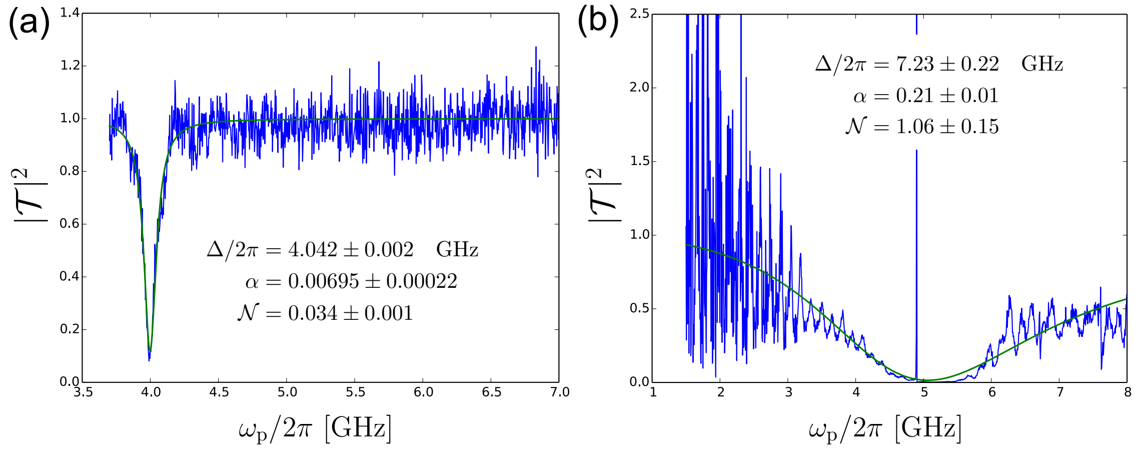

Supplementay Figure 4. Transmission $|\mathcal{T}|^2$ *vs.* probe frequency at the symmetry point ($\Phi_\epsilon = \Phi_0/2$). The results of fit to the transmission data from experiments provide estimates for α, Δ, and $\mathcal{N}$. The analytical expressions in Supplementary Equations (51)-(54) are used. (a) – Cut at the symmetry point of the spectrum of Device I (see Fig. 2d of the main text). (b) – Cut at the symmetry point of the spectrum of Device II (see Fig. 2e of the main text). In both panels, the (fixed) temperature and cutoff frequency are $T = 90$ mK and $\omega_c/2\pi = 65$ GHz, respectively.

Supplementary Note 7: Estimates for the parameters of Device III

Device III is in a coupling regime that does not allow for an analytical evaluation of the kernels entering the expression for the transmission [see Supplementary Equations (29)-(40)]. As a consequence, we are not able to extract via fit to data the parameters that characterize the coupling regime of Device III, as done for devices I and II. Moreover, the spectrum at the symmetry point for the undriven Device III appears almost featureless in the measured range of probe frequencies, as can be seen in Fig. 2(f) of the main text. For these reasons we proceed as follows. First, we compare the data of the transmission in the static case with the simulations, using for the dimensionless parameter $\mathcal{N}$ the value $\mathcal{N} = 8$ which is somewhat in the center of the estimated range $5 \leq \mathcal{N} \leq 10$ (see the Methods section of the main text). We do this for different values of the bare frequency Δ, associating to each value of Δ the coupling α which best reproduces the data. Finally, we use the transmission data of the driven device to choose the value of Δ that best reproduces, with its associated coupling, the V-shape of the transmission as a function of pump power and static bias [see Fig. 3(e) of the main text].

We note that, independent of the value of Δ and of the associated coupling α, to reproduce the measured levels of transmission in the driven case we have to double the value of $\mathcal{N}$ in the simulations, with respect to the corresponding static case. Nevertheless, these variations in $\mathcal{N}$ do not affect much the V-shape of the transmission in the pump power-bias plane, which allows to chose the best value for Δ.

In Supplementary Figure 5 we compare the measured transmission of the undriven device with simulations performed using different values of α. The data used are two perpendicular cuts – at fixed zero bias and at a fixed probe frequency – of the experimental colormap in Fig. 2(f) of the main text. The results are shown for $\Delta/2\pi$ fixed to the value 8 GHz, namely the one which turns out to give the best agreement with the measurements on the driven device (the value used in the main text). The simulations in Supplementary Figure 5 suggest for Device III the rough estimate $\alpha = 0.8 \pm 0.1$.

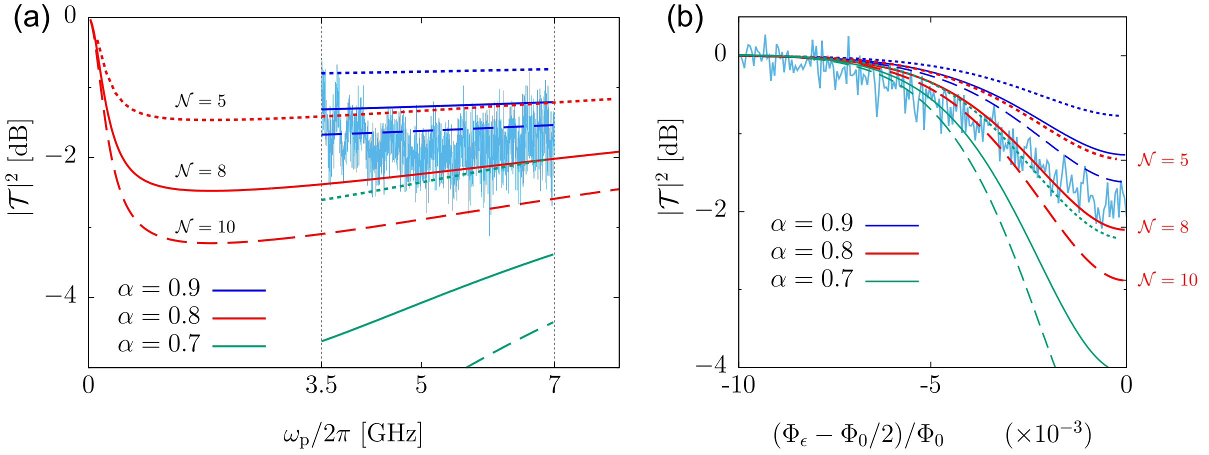

Supplementay Figure 5. Device III – static case. Measured transmission compared with simulations for three values of the coupling α. (a) – Transmission as a function of ω_p at the symmetry point (zero bias). (b) – Transmission as a function of the static bias with fixed $\omega_\mathrm{p} = 5$ GHz. In both panels, three values of $\mathcal{N}$ are shown for each α: $\mathcal{N} = 5$ (dotted lines), $\mathcal{N} = 8$ (solid lines), $\mathcal{N} = 10$ (dashed lines). The plots show that to a larger value of $\mathcal{N}$ there corresponds a larger value of α compatible with the measurements. Simulations are performed by numerically evaluating the kernels [see Supplementary Equations (29)-(40)] with the bath correlation function in the exact scaling limit form of Supplementary Equations (13)-(14). Temperature and cutoff frequency are $T = 90$ mK and $\omega_\mathrm{c}/2\pi = 65$ GHz, respectively.

Supplementary Note 8: Analytical expression for the qubit's response at weak coupling and zero bias

At weak coupling, $\alpha \ll 1$, we can approximate the function $\mathcal{W}(x)$, defined in Supplementary Equation (57), as follows

$$\mathcal{W}(x) = \frac{1}{\alpha + \mathrm{i}\kappa x}\frac{\Gamma(1+\alpha+\mathrm{i}\kappa x)}{\Gamma(1-\alpha+\mathrm{i}\kappa x)} \simeq \frac{1}{\alpha + \mathrm{i}\kappa x}\,. \tag{58}$$

Moreover, at zero static bias, $\varepsilon_0 = 0$, both H^- and $K^-(0)$ vanish. The resulting expression for the linear susceptibility is

$$\chi(\omega_\mathrm{p}) = \frac{H^+(\omega_\mathrm{p})}{\mathrm{i}\omega_\mathrm{p} + K^+(\mathrm{i}\omega_\mathrm{p})}\,. \tag{59}$$

Using the linear response expression (29), which relates $\chi(\omega_\mathrm{p})$ to the transmission $\mathcal{T}(\omega_\mathrm{p})$, we end up with the following approximated expressions

$$\mathrm{Re}\{\mathcal{T}(\omega_\mathrm{p})\} \simeq 1 - \mathcal{N} N_- \frac{\kappa\omega_\mathrm{p} f(\omega_\mathrm{p}) + 2N_+(\kappa\omega_\mathrm{p})^2}{(2\alpha N_+)^2 + f^2(\omega_\mathrm{p})} \;, \tag{60}$$

$$\mathrm{Im}\{\mathcal{T}(\omega_\mathrm{p})\} \simeq \mathcal{N}\frac{N_-}{\alpha} \frac{(\kappa\omega_\mathrm{p})^2 f(\omega_\mathrm{p}) - 2\alpha^2 N_+\kappa\omega_\mathrm{p}}{(2\alpha N_+)^2 + f^2(\omega_\mathrm{p})} \;, \tag{61}$$

where $f(\omega_\mathrm{p}) = \alpha^2\omega_\mathrm{p} + \kappa^2\omega_\mathrm{p}^3 - 2N_+\kappa\omega_\mathrm{p}$.

From Supplementary Equation (59), the imaginary part of the linear susceptibility at zero bias and arbitrary α reads

$$\chi''(\omega_\mathrm{p}) = \frac{\mathrm{Im}\{H^+(\omega_\mathrm{p})\}\mathrm{Re}\{K^+(\mathrm{i}\omega_\mathrm{p})\} - \mathrm{Re}\{H^+(\omega_\mathrm{p})\}(\omega_\mathrm{p} + \mathrm{Im}\{K^+(\mathrm{i}\omega_\mathrm{p})\})}{\mathrm{Re}\{K^+(\mathrm{i}\omega_\mathrm{p})\}^2 + (\omega_\mathrm{p} + \mathrm{Im}\{K^+(\mathrm{i}\omega_\mathrm{p})\})^2} \;. \tag{62}$$

Now, for $\alpha \ll 1$, by using the analytical expressions for H^+ and K^+ with $\mathcal{W}$ in the approximated form given by Supplementary Equation (58), we get

$$H^+(\omega_\mathrm{p}) \simeq \frac{\kappa N_-/\hbar}{\alpha^2 + (\kappa\omega_\mathrm{p})^2}\left(1 - \mathrm{i}\frac{\kappa}{\alpha}\omega_\mathrm{p}\right) \;, \tag{63}$$

$$K^+(i\omega_\mathrm{p}) \simeq \frac{2\alpha N_+}{\alpha^2 + (\kappa\omega_\mathrm{p})^2}\left(1 - \mathrm{i}\frac{\kappa}{\alpha}\omega_\mathrm{p}\right) \;, \tag{64}$$

so that the imaginary part of $\chi(\omega_\mathrm{p})$ acquires the weak coupling form

$$\chi''(\omega_\mathrm{p}) \simeq -\frac{\kappa N_-}{2\hbar\alpha N_+} \frac{\omega_\mathrm{p}\mathrm{Re}\{k^+(\mathrm{i}\omega_\mathrm{p})\}}{\mathrm{Re}\{K^+(\mathrm{i}\omega_\mathrm{p})\}^2 + (\omega_\mathrm{p} + \mathrm{Im}\{K^+(\mathrm{i}\omega_\mathrm{p})\})^2} \;. \tag{65}$$

In the regime considered here, the peak described by Supplementary Equation (65) is narrow and the function $\mathrm{Re}\{K^+(\mathrm{i}\omega_\mathrm{p})\}$ practically constant within its width (roughly measured by $\mathrm{Re}\{K^+(\mathrm{i}\omega_\mathrm{p})\}$ itself). The position of the peak is thus well approximated by the value ω^* obtained upon requiring that $\omega_\mathrm{p} + \mathrm{Im}\{K^+(\mathrm{i}\omega_\mathrm{p})\} = 0$, which yields

$$\omega^* \simeq \frac{\sqrt{2N_+\kappa - \alpha^2}}{\kappa} \;. \tag{66}$$

We remark that this approximate analytical result is valid for the unbiased system, $\varepsilon_0 = 0$, in the limit $\alpha \ll 1$. We did not make use of the above approximated results in the main text. However, they show how, in the weak coupling regime, the response χ'' acquires a Lorentzian shape. Deviations from this Lorentzian behavior are found as α goes beyond the perturbative regime. This can be seen in Fig. 2 (a-c) of the main text and in Supplementary Note 9, where a comparison is made of the qubit response in three different coupling regimes which span the range from weak to ultrastrong coupling.

Supplementary Note 9: Dynamical regimes from transmission for the undriven spin-boson

In the linear (weak probe) regime, the intrinsic properties of the qubit are not influenced by the presence of the probe field. The dynamical behavior of the qubit in absence of driving is fully encoded in the imaginary part χ'' of the linear susceptibility. Specifically, in the underdamped regime, analogously to the damped harmonic oscillator, the position of the peak of χ'' and its full width at half maximum (FWHM) are related to the renormalized oscillation frequency and to the decay rate of the oscillations, respectively. Thus, according to Supplementary Equation (29), by measuring the (real part) of the transmission at weak probe, the imaginary part of the susceptibility χ is accessed which contains the information about the dynamical properties of the static qubit.

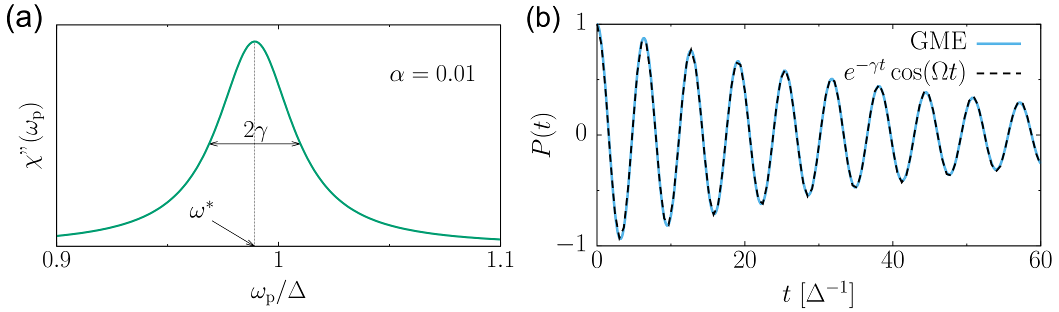

Supplementay Figure 6. Dynamical regime from the susceptibility: Coherent regime. (a) – Imaginary part of the linear susceptibility $\chi(\omega_p)$ (arbitrary units) calculated by means of the analytical expressions in Supplementary Equations (51)-(54). $\chi''(\omega_p)$ has a peak at frequency ω^* with FWHM 2γ. (b) – Comparison between the dynamics obtained from the GME (2) (solid line), with $\varepsilon(t) = 0$ and bath correlation function $Q(t)$ in the exact scaling limit form of Supplementary Equations (13)-(14), and the damped oscillations with renormalized oscillation frequency and decay rate given by $\Omega = \sqrt{(\omega^*)^2 - \gamma^2}$ and γ, respectively (dashed line). Parameters are $\alpha = 0.01$, $T = 0.5\ \hbar\Delta/k_B$, $\varepsilon_0 = 0$, and $\omega_c = 10\ \Delta$.

Consider the case of zero static bias, $\varepsilon_0 = 0$. The imaginary part χ'' of the susceptibility is characterized by a peak centered at a frequency ω^* and of FWHM 2γ. In the coherent regime, occurring when $\omega^* > \gamma$, the dynamics of $P(t)$ displays damped oscillations with renormalized

oscillation frequency $\Omega = \sqrt{(\omega^*)^2 - \gamma^2}$ and damping rate γ. The transition to the incoherent regime is determined by the condition $\omega^* = \gamma$. The incoherent regime, which is realized for $\omega^* < \gamma$, is described by an exponential decay of $P(t)$ with rate γ_r, the relaxation rate, given in this case by the position of the peak.

As an illustration, let us consider the three different dissipation regimes mentioned above,

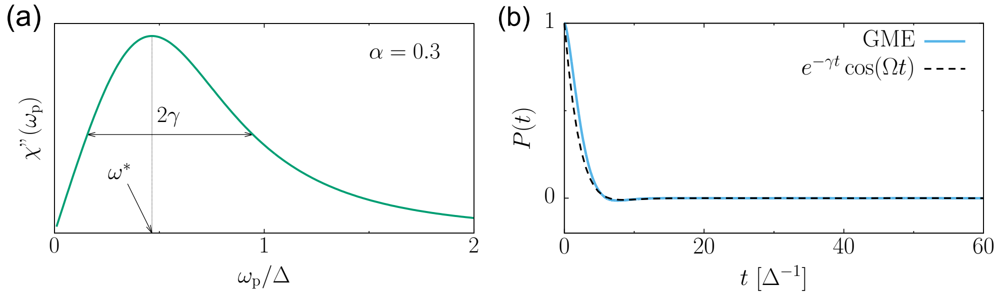

Supplementay Figure 7. Dynamical regime from the susceptibility: Coherent-incoherent transition regime. (a) – Imaginary part of linear susceptibility $\chi(\omega_\mathrm{p})$ (arbitrary units) numerically evaluated by using Supplementary Equations (34)-(40) with bath correlation function $Q(t)$ in the exact scaling limit form of Supplementary Equations (13)-(14). $\chi''(\omega_\mathrm{p})$ has a peak at frequency ω^* of FWHM 2γ. (b) – Comparison between the dynamics obtained from the GME (2) (solid line), with $\varepsilon(t) = 0$ and bath correlation function $Q(t)$ in the exact scaling limit form, and the damped oscillations with renormalized oscillation frequency and decay rate given by $\Omega = \sqrt{(\omega^*)^2 - \gamma^2}$ and γ, respectively (dashed line). Parameters are $\alpha = 0.3$, $T = 0.5\ \hbar\Delta/k_\mathrm{B}$, $\varepsilon_0 = 0$, and $\omega_\mathrm{c} = 10\ \Delta$.

namely i) coherent, ii) coherent-incoherent transition, and iii) incoherent. We calculate by Supplementary Equation (39) the imaginary part of χ as a function of the probe frequency and compare the resulting dynamics, namely damped oscillations or incoherent decay with parameters defined by ω^*, γ, and γ_r, with the dynamics obtained from direct integration of the GME (2) in the static, unbiased case, $\varepsilon(t) = 0$. Results are shown in Supplementary Figures 6-8. On the basis of the considerations made above, we are able to establish a phase diagram for the nondriven spin-boson model, i.e., to assign a dynamical behavior (coherent/incoherent) to the points of the coupling-temperature parameter space, by studying $\chi''(\omega_\mathrm{p})$ and specifically the condition for the coherent-incoherent transition $\omega^* = \gamma$, where ω^* is the position of the peak of

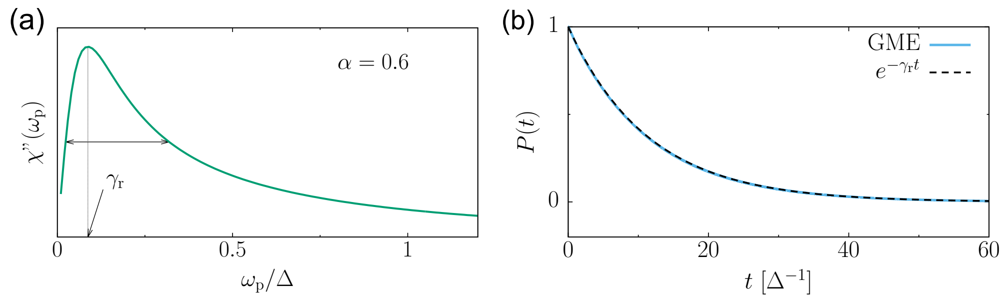

Supplementay Figure 8. Dynamical regime from the susceptibility: Incoherent regime. (a) – Imaginary part of the linear susceptibility $\chi(\omega_p)$ (arbitrary units) numerically evaluated by using Supplementary Equations (34)-(40) with bath correlation function $Q(t)$ in the exact scaling limit form of Supplementary Equations (13)-(14). $\chi''(\omega_p)$ has a peak at frequency γ_r. (b) – Comparison between the dynamics obtained from the GME (2) (solid line), with $\varepsilon(t) = 0$ and bath correlation function $Q(t)$ in the exact scaling limit form, and the exponential decay with rate γ_r (dashed line). Parameters are $\alpha = 0.6$, $T = 0.5\ \hbar\Delta/k_B$, $\varepsilon_0 = 0$, and $\omega_c = 10\ \Delta$.

$\chi''(\omega_p)$ and 2γ its FWHM.

Such phase diagram, derived within the NIBA, is shown in Fig. 1(c) of the main text for $\omega_c = 10\ \Delta$. The curve, representing the transition temperature T^* as a function of α, is an interpolation of the point-set obtained by numerically evaluating $\chi''(\omega_p)$ by means of Supplementary Equations (34)-(40), with the bath correlation function $Q(t)$ in exact scaling limit form in Supplementary Equations (13)-(14), and searching for the coherent-incoherent transition condition $\omega^* = \gamma$. Specifically, fixing the (dimensionless) temperature to the values $k_B T/\hbar\Delta = 2.5,\ 2,\ 1.5,\ 1,\ 0.75,\ 0.5,\ 0.25$, and 0.1, a numerical search for the value of α realizing the condition $\omega^* = \gamma$ was performed. The lowest point, of abscissa $\alpha = 0.5$, is individuated by the exact result $k_B T^*(\alpha = 0.5)/\hbar\Delta = \Delta/2\omega_c$ [1].

Supplementary References

[1] Weiss, U. *Quantum dissipative systems* (World Scientific, Singapore, 4th Ed. 2012).

[2] Leggett, A. J. *et al.* Dynamics of the dissipative two-state system. *Rev. Mod. Phys.* **59**, 1-85 (1987).

[3] Grifoni, M. and Hänggi P. Driven quantum tunneling. *Phys. Rep.* **304**, 229-358 (1998).

[4] Vool, U. and Devoret, M. H. Introduction to quantum electromagnetic circuits. *Int. J. Circ. Theor. Appl.* **45**, 897 (2017).

[5] Grifoni, M., Sassetti, M. Hänggi., P., and Weiss., U. Cooperative effects in the nonlinearly driven spin-boson system. *Phys. Rev. E* **52**, 3596 (1995).

[6] Gradshteyn, I. and Ryzhik, I. *Table of Integrals, Series, and Products* (Academic Press, New York, 7th Ed. 2007).
